# Online Brand Community User Segments: A Text Mining Approach

**DOI:** 10.3389/frai.2022.900775

**Published:** 2022-07-18

**Authors:** Ruichen Ge, Hong Zhao, Sha Zhang

**Affiliations:** School of Economics and Management, University of Chinese Academy of Sciences, Beijing, China

**Keywords:** online brand community, user segmentation, UGC, user churn, text mining

## Abstract

There is a trend that customers increasingly join the online brand community. However, evidence shows that there are nuances between different user segments, and only a small group of users are active. Thus, one key concern marketers face is identifying and targeting specific segments and decreasing user churn rates in an online environment. To this end, this study aims to propose a UGC-based segmentation of online brand community users, identify the characteristics of each segment, and consequently reduce online brand community users' churn rate. We used python to obtain users' post data from a well-known online brand community in China between July 2012 and December 2019, resulting in 912,452 posts and 20,493 users. We then use text mining and clustering methods to segment the users and compare the differences between the segments. Three groups—information-oriented users, entertainment-oriented users, and multi-motivation users—were emerged. Our results imply that entertainment-oriented users were the most active, yet, multi-directional users have the lowest probability of churn, with a churn rate of only 0.607 times than that of users who focus either on information or entertainment. Implications for marketing and future research opportunities are discussed.

## Introduction

Online brand communities provide an interactive platform for companies and consumers (Haverila et al., 2020). Online brand community attracts users who share common interests with a brand (Kuo and Feng, 2013) and allows users to freely communicate, discuss, evaluate, and comment on products (Hajli et al., 2017) and exchange their interests and hobbies, satisfying their information and entertainment needs. For companies, online brand communities play a critical role in increasing customer brand loyalty through relational marketing (Kuo and Feng, 2013). Starbucks Coffee, Dell, and Procter & Gamble are making significant investments in online brand communities in an effort to build stronger relationships with their consumers (Baldus et al., 2015). Companies need to build the loyalty of their users not only to the brand, but also to the community itself (Haverila et al., 2020).

Most prior research assumed that users of online brand communities were homogeneous in terms of behaviors and preferences (Dessart et al., 2019) because users share a common understanding and collective identity (Kuo and Feng, 2013). However, this is not necessarily true (Haverila et al., 2020). Previous research has proven that there is heterogeneity in user tastes on social media (Susarla et al., 2012). We propose that users can engage with the online brand community in different ways (e.g., information or entertainment-oriented). The segmentation is therefore necessary since differences in online brand community users may affect their expectations of the online brand community and how they build loyalty to the brand and the community (Kuo and Feng, 2013). We answer calls for research on “a more diverse classification of participation” (Malinen, 2015, p. 228) of online brand community users.

A small number of previous research (e.g., Shao et al., 2015) has examined the heterogeneity of users in online brand communities, but has mainly focused on the demographic characteristics, access frequency and session duration of community users. The differences between online brand community users lie not only in their general behavior (such as access frequency and session duration), but also in what specific content they focus on, which reflects their inner interests and expectations for the online brand community. The information users create publicly [i.e., User Generated Contents (UGC), Saura et al., 2021a] in the online brand community reflects specific areas they are attracted to. Massive authentic and personalized user-generated content (UGC) (Krumm et al., 2008) generated on social media provides a new possibility for decision-makers to extract customer insights (Moe and Netzer, 2017). By analyzing UGC in different segments, marketers could identify each segment's preference and their covariates, and accordingly, companies could target specific customer groups with content and products appealing to consumers in the segment. As such, firms are able to effectively engage with their customers or online brand community users (An et al., 2018).

In addition, most prior literature in online customer segmentation has focused on using a self-administered survey (Vilnai-Yavetz and Tifferet, 2015). For instance, Underwood et al. (2011) used an online survey to ask Facebook users' personalities, behavior, and activity. They identified three segments: high broadcasters, high communicators, and a high interaction segment. Unfortunately, while self-reports are a valuable means of gathering data in the social sciences (Vilnai-Yavetz and Tifferet, 2015), the method has several pitfalls, such as the difficulty of remembering past behavior (Brewer, 2000) and social desirability bias (De Jong et al., 2010). In contrast, UGC arise from intentional user publications and is the result of user actions in digital environments (Saura et al., 2021a), thus having a higher degree of objectivity. The analysis of these objective data allows companies to better understand user intentions and predict their behavior (Saura et al., 2021a,b), thereby targeting modifications to the information structure of their websites and increasing the likelihood of achieving engagement and user retention rate (Saura et al., 2021a).

In summary, this study is among the first to investigate how objective UGC can be used to explore user heterogeneity in order to build better online brand communities and retain users. First, we classify posts of online brand community users based on Support Vector Machine (SVM) classification, then cluster the users by their posts using the K-means method, and compare the behavioral characteristics of different segments, especially churning behavior. We further used a logistic regression model to investigate user churn rates in different market segments. By doing so, we add to the online brand community segmentation and user churn literature. Practically, this study will help marketers to better understand the online brand community user segments. Thus, it is helpful for the online brand community to reach a broad spectrum of users efficiently (Bulut and Dogan, 2017), and design different strategies and practices accordingly to improve retention rates for different user segments (Bulut and Dogan, 2017).

In sum, our research questions are as follows:

RQ1: How online brand community users could be segmented according to their posts?

RQ2: How do these different types of users differ by the meaning of behavior characteristics, especially churning behavior?

## Materials and Methods

### Dataset

We collected data from Pollen Club (club.huawei.com), a large online brand community owned by Huawei Technologies Co., Ltd. Users could seek and share information on Pollen Club, such as their opinions and suggestions about the product and problems in the use of the product. At the same time, the online forum also includes social entertainment functions, where users could exchange interests and hobbies (e.g., sharing photos taken by Huawei phones). This research focused on “Huawei Watch,” a sub-section post area in Pollen Club, and we crawled all posts and user pages in this section from July 2012 to December 2019. After data cleansing, our data set contained 912,452 posts and 20,493 users. Each post contains information on the user name, the date of the post, and its text. Each post is linked to its author's home page so that we can obtain user information variable, such as the number of friends, the number of posts and replies, popularity (measured by the number of fans followed one's posts), and prestige (calculated by the number of one's posts are highlighted by forum administrator).

Additionally, we paid particular attention to user churn. Churn is defined as the loss of a user in an online social network (Long et al., 2012). Users are annotated to be churn or non-churn by examining login activities to the site at some time in the future (Long et al., 2012). According to our preliminary survey of users on Pollen Club, churners in this study were defined as the users who haven't made any login or activity record in Pollen Club for the last 3 months.

### Text Classification

Machine learning and natural language processing algorithms are used to analyze the massive amount of textual social media data available online (Albalawi et al., 2020), including text classification techniques. Text classification is a method used to confirm the category of an unlabeled text based on the defining topic categories in advance (Miao et al., 2018). It is a supervised learning approach in which a training set of documents {D1, D2….Dn} labeled with classes from {1…m} is used to build a classification model and predicts the class label of a new incoming document based on the training model (Vijayan et al., 2017). Support vector machines are linear classifiers suitable for classifying high-dimensional data (Altinel et al., 2015; Thangaraj and Sivakami, 2018). Its main idea is: for a multidimensional sample set; each sample is represented as a point in space. Then the system randomly generates a hyperplane that continuously moves and classifies the samples until the points belonging to the same class are completely distributed on the same side of the hyperplane. There are many hyperplanes that satisfy this condition, and we need to find such a plane that maximizes the blank area between its edges to achieve the optimal classification of these samples. For the new data, we map it to the same space and predict the category based on its location (Miao et al., 2018).

SVM performs well in text classification scenarios because the vectorized representation of text involves a high-dimensional feature space (Vijayan et al., 2017). SVM also performs with the same accuracy even when the data is sparse (Thangaraj and Sivakami, 2018). Therefore, SVM could exert its effectiveness in short text classification, which has been proved in existing studies. Almost all user posts on social media sites are short texts. For instance, Yin et al. (2015) show that SVM can classify a large number of short texts to mining the useful massage from the short text. Wang et al. (2017) successfully categorize labeled short text documents in Chinese using kernel SVM as the classifier, and their results show that the SVM method outperforms other conventional classification methods such as k-Nearest Neighbor and Decision Tree. In sum, SVM has been widely used in the short text classification of social media sites (Yin et al., 2015; Hu et al., 2018).

**Figure 1:**
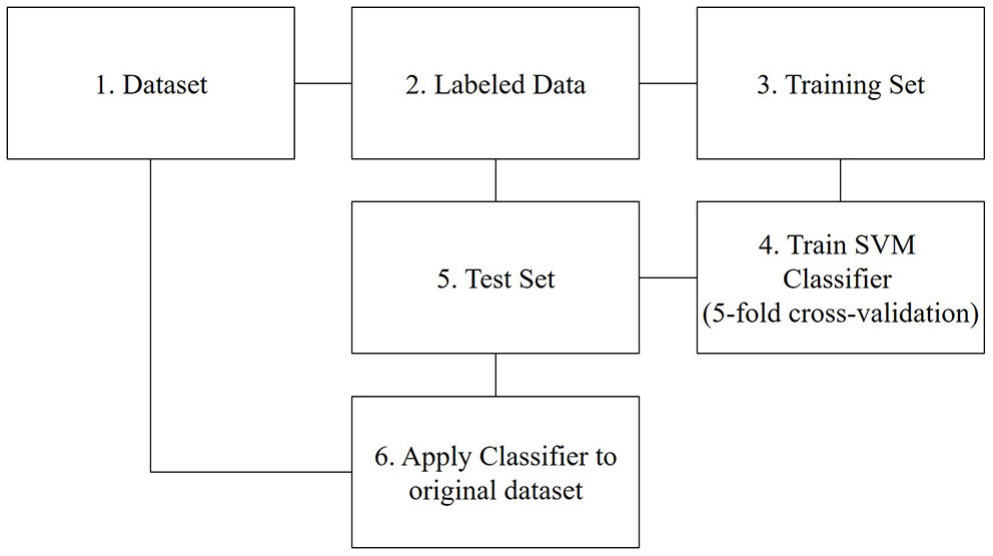
The steps of data classification.

According to the above reasons, we chose the SVM method to classify the posts. Before we classified the posts, we pre-processed the collected posts, including two steps. First, we utilized the Chinese word division procedure. Compared with English words, there are no spaces between Chinese words. Hence, we must do a word segmentation operation on the Chinese short text as the first step (Yin et al., 2015; Wang et al., 2017). We used the “jieba” package on Python to split the words. Some words occur frequently without useful meaning, are called “deactivated words,” such as “because,” “so,” “although,” “but,” etc. We removed these deactivated words to ensure the classification effect. Second, we conducted text representation. We used the term frequency (TF)-inverse document frequency (IDF) model, which reflects the importance of a word for a document in a dataset (Wang et al., 2017). As such, we transformed Chinese documents into structured forms. Lastly, we followed the steps depicted in Figure 1 and described below to divide all posts into two categories: informational posts and entertaining posts. Typical informational posts refer to users exchanging information about (Huawei) products (see e.g., 1 and 2), where users share their interests and life, are classified as entertaining posts (see e.g., 3 and 4). We also hired three assistants to label the posts. They first gave an overview of all posts, summarizing the characteristics of both types of posts. They were then asked to label the posts independently. To reduce personal bias, we used a majority voting strategy and the final labeling was the result of a majority agreement.

e.g., 1: The sports log of the Huawei Watch is inaccurate. How can I adjust it?

e.g., 2: You could go to the Huawei service center to upgrade the tablet's memory for free to reduce the latency.

e.g., 3: It's a nice day today. I went to the lake with my daughter and took some photos. My daughter is so pretty!

e.g., 4: Today, I successfully challenged half marathon for the first time. I feel very tired. I need more exercise. Come on!

Step 1: We use all posts from our dataset (*N* = 912,452)

Step 2: Following the word division method in Rietveld et al. (2020), we draw a stratified sample to create a representative set of 45,622 posts (~5% of the dataset) and coded them as described above.

Step 3: We split the data in training (80%) and test set (20%). 5-fold cross-validation was used to train the model to enhance the model performance (Asrol et al., 2021). The labeled data is split into five subsets of identical size, one of these subsets is retained as the test dataset, and the rest of the subsets are utilized as the training data set. The operation is replicated five times, and each subset is used exactly once to test the model. The results from these replicates are merged into a single estimate (Ramírez-Correa et al., 2021).

Step 4: Based on the SVM model, we trained a text classifier using the training data.

Step 5: We used the trained model to assess the performance of our model using the test set.

Step 6: The remaining data (without the training and test set) is classified using the trained SVM classifier. 492,354 posts were coded as informational posts (53.98%), and 419,918 posts were coded as entertaining posts (46.02%), indicating a balanced dataset.

The correctness of a classification can be evaluated by computing the number of correctly recognized class examples (tp), the number of correctly recognized examples that do not belong to the class (tn), and examples that either were incorrectly assigned to the class (fp) or that were not recognized as class examples (fn) (Sokolova and Lapalme, 2009). In order to compare the classification accuracy of those three methods, we chose Accuracy, Precision, Sensitivity and Specificity as evaluation standards, which are commonly used methods to assess the performance of binary classifiers (Sokolova and Lapalme, 2009). Table 1 shows the measures' calculation formula and their performance in our study.

**Table 1:** Measure and performance of SVM.

**Measure**	**Formula**	**Performance**
Precision	$\frac{tp}{tp+fp}$	0.84
Accuracy	$\frac{tp+tn}{tp+fp+tn+fn}$	0.84
Sensitivity	$\frac{tp}{tp+fn}$	0.83
Specificity	$\frac{tn}{tn+fp}$	0.85

### User Segmentation

In the previous section, all posts were classified as either informational or entertaining. We then counted the number of posts each user made in both of the two categories. In particular, we generated a new variable called “diversity,” which is a binary variable and assigns it a value of 1 if the user wrote both the information and entertainment posts and 0 otherwise.

We used K-means, one of the most well-known clustering algorithms, to cluster users. K-means is a simple unsupervised learning algorithm, used to classify data based on Euclidian Distance technic between the data (Jamadar and Loni, 2016). This algorithm divides data into k sections and computers randomly select and assign objects to one cluster (k). The distance between each object and the center of each cluster is calculated and resulted in an optimal cluster solution (Marutho et al., 2018). The K-means clustering procedure is less susceptible to outliers in the data (Hair et al., 2003), and it is commonly used in marketing segmentation research (Shao et al., 2015). For instance, Foster et al. (2011) apply the K-means approach to identify user clusters on social media. Similarly, Alsayat and El-Sayed (2016) use the K-means clustering algorithm to group user communities according to their activities on social media sites. Thus, we chose K-means for the user cluster because it performs well in an online social media context.

## Results

### Comparison of Differences Between the Segments

Took the number of informational posts, the number of entertaining posts, and diversity as the input variables, we used the K-means algorithm to cluster users, and the elbow method was used to select the optimal number of clusters (Marutho et al., 2018). Finally, we determined the number of clusters (K) to be 3, in other words, users were divided into three categories. Table 2 shows the cluster center coordinate values of the three types of users, reflecting the average performance of each segment on each attribute.

**Table 2:** Centers of three clusters.

**Attribute**	**Segment 1**	**Segment 2**	**Segment 3**
Number of informational posts	0.98	0.13	0.72
Number of entertaining posts	0.07	0.97	0.58
Diversity	0.03	0.04	0.24

Segment 1 prefers making informational posts to entertaining posts, while Segment 2 tends to create more entertaining posts compared with informational posts. Both Segment 1 and Segment 2 have a low score on the dimension of diversity. Segment 3 has a high score on all three dimensions, which means that they access Pollen Club both to gather information and to find entertainment. Therefore, we identified Segment 1 as “Information-oriented users,” Segment 2 as “Entertainment-oriented users,” and Segment 3 as “Multi-motivation users.”

Table 3 shows a comparison of the characteristics of each segment. Compared with information-oriented users and multi-motivation users, entertainment-oriented users top in every dimension such as the number of friends, posts, replies, popularity, and prestige. Information-oriented users are relatively passive, with the lowest desire to share and communicate among all the identified segments. However, these two segments have similar churn rates (churn rate of information-oriented users = 93%, the churn rate of entertainment-oriented users = 94%). Multi-motivation users have the lowest probability of churning (churn rate = 91%), significantly lower than the other two segments (*F* = 27.57, *p* < 0.001), although they are less active than entertainment-oriented users.

**Table 3:** Characteristics of each segment.

**Characteristics**	**Information-oriented users**	**Entertainment-oriented users**	**Multi-motivation users**
Number of posts	27.31	175.77	61.74
Number of replies	664.07	2219.33	1497.02
Number of friends	4.69	8.27	7.83
Popularity	620.11	2203.04	1218.90
Prestige	68.35	118.15	103.91
Churn rate	0.93	0.94	0.91
Size of Cluster	10487	4000	6006

### User Churn in Different Segments

In order to ensure the robustness of the results, we further used a logistic regression model to investigate user churn rates in different segments. Our purpose is to determine to what extent diversity influences user churn. Because the dependent variable churn is binary, we use a logit model, which is widely used in customer churn research (De Caigny et al., 2018). To better estimate the impact of diversity, we run two different models. Model 1 is the controls-only model. We consider the effect of the number of information and entertainment posts, the number of friends, popularity, and prestige in this model. Then, we added the main independent variable diversity to the model (Model 2), as shown in equation (1).


(1) $$\begin{array}{l} Churn_{i}=\beta_{0}+\beta_{1}Diversity_{i}+\beta_{2}Information_{i}\\ \;+\beta_{3}Entertainment_{i}+\beta_{4}Friends_{i}\\ \;+\beta_{5}Popularity_{i}+\beta_{6}Prestige_{i}+\tau_{i} \end{array}$$

Correlation analysis of the variables showed that the correlation coefficient between the variables was below 0.5 (the maximum correlation coefficient is 0.43) and there was no serious multicollinearity. Depicted in Table 4 are the estimation results of our regression analyses. As can be seen, the Pseudo *R*^2^ increases from 14.3 to 14.8%, suggesting that the inclusion of diversity increases the explanatory power of the model (Majumdar and Bose, 2018). Meanwhile, the addition of diversity improved the model fit (*F*-test χ^2^ = 46.14, *p* < 0.001). The negative significance of diversity (β = −0.248, *p* < 0.001) implies that users who focus on both informational content and entertaining content are less likely to churn, with a churn rate of only 0.607 times than that of users who focus on a single type of content. In addition, we found that the number of informational posts could decrease churn significantly (β = −0.087, *p* < 0.01), while the number of entertaining posts had no significant effect on churn (*p* > 0.1). For every increase in the informational posts made by one user, his/her probability of churn decreases by 0.1%.

**Table 4:** Regression results.

**Variables**	**Model 1**		**Model 2**	
	**Coeff**	**OR**	**Coeff**	**OR**
Diversity			−0.248***	0.607
No. of posts of Entertainment	−0.019	1.000	0.035	1.035
No. of posts of Information	−0.103**	0.999	−0.087**	0.999
No. of friends	0.004	1.000	−0.006	1.006
Popularity	0.197*	1.000	0.329*	1.391
Prestige	−0.061*	0.999	−0.068*	0.934
Year Dummy	-		-	
Pseudo *R*^2^	0.143	0.148
Log Likelihood	−3979.87	−3955.38
No. of observations	20,493	20,493

*p <0.1; **p <0.01; ***p <0.001. Coeff, Standarized Coefficients; OR, Odds Ratio.

## Discussion

### Conclusion

Online brand communities provide a platform for deeper interactions with customers (McLaughlin and Davenport, 2017). As the volume of consumers using the online brand community continues to grow (Campbell et al., 2014), research attention has been paid to the segmentation of the online brand community (Barnes et al., 2007). The online consumer segmentation has been investigated from many different perspectives (Shao et al., 2015). We propose a UGC-based segmentation of users of the online brand community.

Specifically, we cluster the users by their posts and investigate the behavioral differences between different types of users. On the one hand, we find that online brand community users fall into three distinct segments with significant differences in user behavior: information-oriented users, entertainment-oriented users, and multi-motivation users. The user segments that we named information-oriented users predominantly use the online brand community to gather information, which is similar to “finders” named by Shao et al. (2015). Entertainment-oriented users have similar features with “socializers” of Shao et al. (2015)'s identification and mainly use the online brand community to satisfy entertainment needs. Multi-motivation users correspond to the “advanced users” of Bulut and Dogan (2017) and Devotees of Shao et al. (2015), who access online communities with high frequency and for long periods of time to gather information and find entertainment.

On the other hand, the result suggests that while entertainment-oriented users are the most active, multi-motivation users have the lowest probability of churn. Using logistic regression, we also confirm that users who focus on both informational content and entertaining content are less likely to leave the online brand community, with a churn rate of only 0.607 times than that of users who focus on a single type of content. Consistent with previous research which shows that there is a high churn rate of participants in online brand communities who emphasize only product-related discussions (Dholakia and Vianello, 2009), we further suggest that users who concentrate on both informational content and entertaining content are more likely to be retained in the online brand community.

### Theoretical Implications

We contribute to online brand community literature in the following ways. First, we challenge the user homogeneity assumption in the online brand community by empirically identifying three user segments. Existing research preassumes that online brand community users share a common consciousness, rituals, and traditions, suggesting homogeneity in the brand communities (Haverila et al., 2020). However, our results reveal that there is heterogeneity in the membership of brand communities. The findings of this study add to the burgeoning heterogeneity view of online brand communities (Susarla et al., 2012; Haverila et al., 2020).

Second, our results underscore the importance of encouraging diversity in the online brand community. Previous studies report that a community with different characteristics would satisfy the distinct needs of consumers (Pan et al., 2014). Moreover, previous studies show that expressive freedom is critical to retain customers within a community (Almeida et al., 2013; Dholakia and Vianello, 2009). Our study extends the research on expressive freedom by highlighting the importance of diverse expression in building a vibrant online brand community.

Third, we are among the first to use a UGC-based segmentation of the online brand community users. Some of the previous studies use a variety of psychographic variables (Underwood et al., 2011; Shao et al., 2015) based on the self-reported surveys to segment online brand community users. Other studies have developed behavior-based online consumer market segments by focusing on different uses of the Internet (Jansen et al., 2011; An et al., 2016, 2018; Zhang et al., 2016). UGC-based segmentation is important because content created by users could reflect their psychological needs (Shen et al., 2016). Previous motivation-based studies found that both entertainment and information seeking were the primary reasons for using social network sites (Kilian et al., 2012; Bulut and Dogan, 2017) and our UGC-based segmentation supports this finding.

### Practical Implications

From a managerial point of view, the current research suggests that different user segments have distinct needs. Entertainment-oriented users expect high entertainment value, whereas information-oriented users predominantly use the online brand community to gather information. Marketers should improve both the entertainment and information value of the online brand community to engage with the needs of different groups. Furthermore, this study suggests online brand communities should actively promote diversified use of online brand communities. Many online brand communities fail because companies emphasize product-related discussions, which leads to consumer participation for functional reasons, without forming bonds or relationships (Dholakia and Vianello, 2009). Marketers may use both informational and entertaining content to reach users. Specifically, in addition to providing information-oriented users with more comprehensive advice on product usage and problem-solving, marketers can also provide incentives to guide them to try entertainment services, such as increasing the hedonic characteristics of a website page or campaign. Similarly, opportunities to create and share both entertainment-oriented content such as users' interests and life as well as information-oriented content such as suggestions and opinions of products could be provided simultaneously. Finally, marketers should encourage users to express themselves freely beyond product discussions in the online brand community. This leads to consumer participation for not only functional reasons, but also intrinsic and social reasons (Dholakia and Vianello, 2009).

### Limitations and Future Research

The limitations of this study suggest avenues for future research. First, we focused on only Pollen Club and consumer behaviors may differ in other online brand community. Further studies should validate user segments among different online brand communities. Second, this study doesn't take cultural differences into consideration. Comparing user segments in other regions could be an interesting direction for future research. Last but not the least, due to data limitations we are unable to investigate the psychological reasons behind our results. For instance, why does diversity not depth reduce user churn rate? We suggest future research to explore the underlying psychological mechanism.

## Data Availability Statement

The raw data supporting the conclusions of this article will be made available by the authors, upon reasonable request.

## Author Contributions

RG, HZ, and SZ: conceptualization. RG and SZ: methodology. RG: resources, formal analysis, visualization, and writing—original draft preparation. HZ and SZ: investigation, supervision, funding acquisition, and writing—review and editing. All authors contributed to the article and approved the submitted version.

## Funding

This work was supported by the National Natural Science Foundation of China (Grant No. 71772169, 71972175, 72172146); and the Fundamental Research Funds for the Central Universities (Grant No. Y95402AXX2).

## Conflict of Interest

The authors declare that the research was conducted in the absence of any commercial or financial relationships that could be construed as a potential conflict of interest.

## Publisher's Note

All claims expressed in this article are solely those of the authors and do not necessarily represent those of their affiliated organizations, or those of the publisher, the editors and the reviewers. Any product that may be evaluated in this article, or claim that may be made by its manufacturer, is not guaranteed or endorsed by the publisher.
